
JOURNAL INFORMATION
==============================
NLM Title Abbreviation: Atlanta Med Surg J (1884)
NLM Journal ID: 101251623

Atlanta Medical and Surgical Journal (1884)

Publisher: Biomedical Journal Digitization Project

ARTICLE INFORMATION
==============================
PMCID: PMC9201854
PMID: 35829606
Article version: 1
Subjects: Original Communications

Suprapubic Cystotomy for Stone, with Report of Two Interesting Cases* President’s Annual Address before the Atlanta Society of Medicine, January 5, 1899.

Goldsmith Wm. S. M.D. Atlanta, Ga.; Lecturer on Genito-urinary Diseases and Syphilis, Atlanta College of Physicians and Surgeons

Print publication date: 1899 Feb
Volume: 15
Volume Series: New Ser
Issue: 12
First page: 793
Last page: 797
License: This work is free of known copyright restrictions. For more information, please see PMC Back Issue Digitization.
License URL: https://creativecommons.org/publicdomain/mark/1.0/

==============================

ATLANTA

### Medical and Surgical Journal.

Vol. XV. FEBRUARY, 1899. No. 12.

DUNBAR ROY, A.B., M.D., EDITOR.

M. B. HUTCHINS, M.D., BUSINESS MANAGER.

### *ORIGINAL COMMUNICATIONS.*

### SUPRAPUBIC CYSTOTOMY FOR STONE, WITH REPORT OF TWO INTERESTING CASES.*

By WM. S. GOLDSMITH, M.D.,

Lecturer on Genito-urinary Diseases and Syphilis, Atlanta College of Physicians and Surgeons.

The operation of suprapubic cystotomy is of sufficient age to entitle it to an honorable antiquity, though, in common with other operations of merit, has suffered lapses, during which time other methods for the removal of vesical calculi have been introduced. Pierre Franco, in 1556, first performed this operation as a means of extricating himself from an embarrassing dilemma. An unsuccessful attempt to remove a stone from a child by the perineal route induced him to open the abdomen and explore the bladder from above. The patient recovered, but the surgeon condemned the method. The real inventor of the operation was Roussetus, who never performed it. In 1590 he published a work on Cæsarean section, in which was given an elaborate and accurate descrip-

*President's Annual Address before the Atlanta Society of Medicine, January 5, 1899.

tion of all the pelvic organs. He described minutely the principles and technique of suprapubic cystotomy, and, among other things, corrected the prevailing idea that wounds of the bladder were necessarily fatal.

At this period the operation was performed in an experimental manner by many surgeons, but, owing to the meager literature on the subject and its slight encouragement, nothing was done towards its advancement until the Douglas revival in 1718. Thornton of Bristol, indorsed the procedure, and in 1727 wrote a valuable treatise, in which he detailed an account of fifteen cases. He was the first author to direct attention to the importance of the insertion of the urachus in the bladder as a landmark in outlining the boundary of the prevesical space and in determining the presence of the peritoneum. This guide, as relating to the latter, is not altogether infallible, as occasionally the peritoneum folds around the urachus, dipping down on each side, and almost completely obliterating this space. The subsequent history of the operation shows a steady decline in favor. The advent of Listerism and the tremendous impetus given by Garson and Petersen, upon the introduction of rectal distention as a means of elevating the bladder to the extent of lengthening the suprapubic space, placed the method upon a firm foundation, which has broadened and developed into a position approaching the ideal. The position and condition of the bladder are important elements to be considered in the technique of the operation. In children and early adult life, we are favored by the high position of the organ, and by its usual good condition. As age advances, the bladder descends lower in the pelvis, and structural changes are much more likely to occur. Such circumstances contribute largely, of course, to the favorable termination of the case, and statistics bearing upon this subject should be based upon classifications of periods of ten years of the patient's age.

I shall not, at this time, take up the relation of hypertrophied prostate to stone in the bladder, a theme in itself fertile and fascinating. In this connection I believe every one will agree that the extraction of the stone will, in a great measure, remove the cause of the hypertrophy and conduce, if the necessity arises, to the rapid and easy familiarity of catheter life. Rectal distention, as one of the accredited agents looking to the revival of this operation, has had many words of commendation and not a few of condemnation. The majority of operators have abandoned its use, and the minority, but for force of habit, could do as well without it. There is really very little to be gained by rectal distention, as practically the same results can be obtained by other aids, simple and easy of application. The distention of the bladder with air, as suggested by Bristow, and further elaborated by Tilden Brown, is practiced to some extent in the North. To my mind the ideal distention is by a fluid medium, antiseptic in character. Boro-clyceride or the normal salt solution are admirable agents. Not a few surgeons prefer to operate without any kind of distention, either vesical or rectal, utilizing the Trendelenburg position, claiming that increased operating area more than offsets the inconvenience of non-distention.

The question of immediate closure of the bladder in selected cases is of much interest, since its adoption tends to greatly shorten the period of convalescence and gives us a closed cavity which drains, as nature intended, through the urethra. The cases to which immediate and complete suture is applicable are many, much greater in fact than the average man accepts. I think every bladder, except those containing embedded stones and those of the ulcerative class, should be closed tight. One of the objections to this method occurs in old men, in whom the bladder is very low in the pelvis, the walls thickened and its cavity greatly lessened. If this particular type is not closed, certainly the organ should be anchored in such a manner that the opening for drainage is fixed and staple. It is reasonable, therefore, that if the incision is accurately sutured, union will almost certainly occur, provided proper urethral drainage is secured. In one of the cases I shall briefly discuss this evening good union obtained, in spite of a wound practically lacerated in character. The bladder walls were so friable that in making slight traction with catch forceps pieces of the entire wall would be torn away. In my other case, an ideal one for immediate closure from the standpoint of age, position and condition of bladder, I was forced to anchor and drain on account of oozing and extensive traumatism incident to the removal of an embedded stone. Having outlined the principles and expressed an opinion on some of the mooted points connected with this operation, I shall report, without detail, two recent cases.

Male, aged 51, came to me the latter part of November for operation for hemorrhoids. General condition fair, but the rectum was so irritable that a week's local treatment and rest were advised. The hemorrhoids began to appear twenty years ago, and as usual every suggested remedy was tried. The bladder symptoms appeared five years ago and had grown steadily worse. I suspected stone and made several incomplete explorations, but excessive irritation prevented a satisfactory examination. The urine was loaded with mucus, very little pus, albumen, tube casts and innumerable bacteria. This combination of circumstances for the moment brought matters to a standstill. Finally, on December 4th, from sheer necessity, I operated on the hemorrhoids. While under ether, the stone-searcher confirmed my fears, and one week later I removed two stones. I closed the bladder with silk, using the mattress suture. The abdomen was also closed with silk, except at the lower angle, where I left a small opening for drainage. Some provision for drainage should always be made, when the bladder is closed. I was fearful of leakage in this case, which occurred on the seventh day upon the removal of the catheter for a few hours. The replacement of the catheter and its retention for an additional week, gave ample time for a firm union to occur. The bladder was irrigated daily for two weeks with a 1 to 6000 permanganate of potash solution. Subsequent to the withdrawal of the catheter, irrigations every third day have been practiced. The interesting feature of the case is this: that oftentimes the reflexes from the rectum, particularly incident to hemorrhoids, are directed to the bladder and deep urethra, thus making and distorting symptoms of grave conditions in one or both localities.

The second case is that of a male, aged 23. Complained of bladder trouble from early childhood. For the past six years has had from two to four attacks of subacute cystitis a year. He came to me on the 18th of December; general condition poor. He lived in South Georgia and, of course, in a malarial section. I put him on quinine and salol upon arrival, and advised the free administration of Lithia water and lemonade. Nourishing and stimulating diet was instituted. Yielding to the most pitiful importunities for relief, I operated on the 24th. A stone firmly embedded in the base of the bladder was discovered, and a tedious and prolonged effort required for its removal. The condition of the vesical mucous membrane, after the necessarily harsh manipulation, did not warrant the complete closure of the bladder. I sutured the upper half of the incision and anchored the lower half to the abdominal wall, passing my suture through the muscular coat of the bladder and carrying it through the recti and skin. A wick of sterile gauze was inserted to the bottom of the cavity and a catheter fastened in the urethra. I believe in using drainage both ways, as there can never occur such a thing as too much drainage. The catheter was retained only twenty-four hours, on account of urethral irritation. At the same time a rubber drainage tube was substituted for the gauze. Reaction from the operation was rapid and satisfactory and no untoward symptoms occurred until the third day, when marked malarial toxemia was manifest. A temperature of 105 was brought down to 102 by calomel and rectal enemata and the infusion of one and one-half quarts of normal salt solution. Quinine, iron, arsenic by the mouth, hypodermics of strychnine, and daily infusions of the salt solution, together with liberal quantities of alcohol, prolonged the life of the patient until the sixth day, when he died. This case illustrates the peculiar disposition of malaria and the deadly toxin involved. Nothing could have arrested this poison and no one could have anticipated the result.

The mortality of suprapubic cystotomy is about nine per cent., and when we consider that the most aggravated cases are subjected to this operation, it is another evidence of its safety and simplicity.

---

**NOTICE.—This JOURNAL and THE INTERNATIONAL JOURNAL OF SURGERY one year for $1.00 cash.**
